# Occurrence and Molecular Study of Hypermucoviscous/Hypervirulence Trait in Gut Commensal *K. pneumoniae* from Healthy Subjects

**DOI:** 10.3390/microorganisms11030704

**Published:** 2023-03-09

**Authors:** Dina M. Osama, Bishoy M. Zaki, Wafaa S. Khalaf, Marwa Yousry A. Mohamed, Mahmoud M. Tawfick, Heba M. Amin

**Affiliations:** 1 Department of Microbiology and Immunology, Faculty of Pharmacy, October University for Modern Sciences and Arts (MSA), Giza 12451, Egypt; dmosama@msa.edu.eg (D.M.O.); bzaki@msa.edu.eg (B.M.Z.); htmagdy@msa.edu.eg (H.M.A.); 2 Department of Microbiology and Immunology, Faculty of Pharmacy (Girls), Al-Azhar University, Cairo 11751, Egypt; wafaa.khalaf@azhar.edu.eg; 3 Biology Department, Faculty of Science, Kingdom of Saudi Arabia, Imam Mohammad Ibn Saud Islamic University (IMSIU), Riyadh 11623, Saudi Arabia; mymohamed@imamu.edu.sa; 4 Department of Microbiology and Immunology, Faculty of Pharmacy (Boys), Al-Azhar University, Cairo 11751, Egypt; 5 Department of Microbiology and Immunology, Faculty of Pharmacy, Heliopolis University, Cairo 11785, Egypt

**Keywords:** *K. pneumoniae*, virulence factors, hypermucoviscosity, hypervirulent, gut commensals

## Abstract

Hypervirulent *Klebsiella pneumoniae* (hvKp) is emerging worldwide. Hypermucoviscousity is the characteristic trait that distinguishes it from classic *K. pneumoniae* (cKp), which enables Kp to cause severe invasive infections. This research aimed to investigate the hypermucoviscous Kp (hmvKp) phenotype among gut commensal Kp isolated from healthy individuals and attempted to characterize the genes encoding virulence factors that may regulate the hypermucoviscosity trait. Using the string test, 50 identified Kp isolates from healthy individuals’ stool samples were examined for hypermucoviscosity and investigated by transmission electron microscopy (TEM). Antimicrobial susceptibility profiles of Kp isolates were determined using the Kirby Bauer disc method. Kp isolates were tested for genes encoding different virulence factors by PCR. Biofilm formation was assayed by the microtiter plate method. All Kp isolates were multidrug-resistant (MDR). Phenotypically, 42% of isolates were hmvKp. PCR-based genotypic testing revealed the hmvKp isolates belonged to capsular serotype K2. All study Kp isolates harbored more than one virulence gene. The genes *magA* and *rmpA* were not detected, while the *terW* gene was present in all isolates. The siderophores encoding genes *entB* and *irp2* were most prevalent in hmvKp isolates (90.5%) and non-hmvKp (96.6%), respectively. hmvKp isolates harbored the genes *wabG* and *uge* with rates of 90.5% and 85.7%, respectively. The outcomes of this research highlight the potential health risk of commensal Kp to cause severe invasive diseases, owing to being hmvKp and MDR, and harboring multiple virulence genes. The absence of essential genes related to hypermucoviscosity such as *magA* and *rmpA* in hmvKp phenotypes suggests the multifactorial complexity of the hypermucoviscosity or hypervirulence traits. Thus, further studies are warranted to verify the hypermucoviscosity-related virulence factors among pathogenic and commensal Kp in different colonization niches.

## 1. Introduction

The human gut microbiota comprises diverse microbial groups, including symbiotics, commensal bacteria, and opportunistic pathogens; they have roles in human health and disease conditions. Although it is not a primary gut colonizer like *E. coli*, *K. pneumoniae* (Kp) is one of the most common species that permanently colonize the gut of humans [1]. Kp is a ubiquitous Gram-negative, non-motile bacterium belonging to the Enterobacteriaceae family. Despite its distribution in the environment, the human gut is usually the main reservoir of Kp [2,3]. Further, many studies have markedly emphasized gut Kp as a significant factor in developing gastrointestinal diseases or their progression [4,5,6]. As an opportunistic pathogen, Kp can cause various infections, which may be associated with severe complications, particularly in older and/or immunocompromised patients [5,6,7].

Infections caused by Kp have been classified into hospital-acquired infections (or nosocomial infections) in hospitalized patients and community-acquired infections in healthy individuals [8]. The causative strains of nosocomial diseases are called classical *K. pneumoniae* (cKp). On the other hand, community-acquired infections are believed to be caused by hypervirulent *K. pneumoniae* (hvKp). Significantly, hvKp was identified among the human gut commensal bacteria, which likely enhances its dissemination in communities and healthcare settings [1,9]. The cKp can cause various infections, including bacteremia, pneumonia, urinary tract infections, and gut and soft tissue infections [10,11]. The hvKp was first identified in Taiwan in the 1980s in a clinical case of tissue-invasive Kp infection of an immunocompetent individual [12,13]. Then, hvKp has spread globally from Taiwan to other countries worldwide, including those in America, Europe, Australia, and the Middle East [10]. hvKp can remarkably cause severe invasive metastatic infections. The hvKp-caused invasive infections include pyogenic liver abscesses with a complication of sepsis, endophthalmitis, intraparenchymal disease of the lungs and empyema, meningitis and brain abscess, endophthalmitis, necrotizing fasciitis, and osteomyelitis [1,3,8]. In addition to invasive infections, hvKp strains can cause severe complications in immunocompromised and healthy individuals [13].

Globally, cKp infections are usually MDR, while hvKp-caused conditions are likely susceptible to various antimicrobials. Thus, cKp infections are of considerable clinical significance owing to their resistance to multiple antimicrobial drugs [13]. Many previous studies from Egypt revealed a high incidence of MDR cKp infections in Egypt [11,14,15,16,17,18]. However, hvKp-caused infections are increasingly reported with increased morbidity and mortality rates worldwide [16]. Further, MDR isolates of hvKp have been increasingly detected in clinical settings, harboring different antimicrobial resistance encoding genes [19,20]. The combination of the MDR and hypervirulence phenotypes can create the ultimate superbug, especially as they are among the human gut commensal, so consequently can cause a clinical crisis [19,21].

Kp can express a variety of virulence factors, including capsules, siderophores, iron-scavenging systems, adhesins, and endotoxins [22]. The virulence of the hvKp has been attributed to having higher production levels of capsular or extracapsular polysaccharides than that of cKp, in addition to a more efficient iron acquisition system [13,16,23]. Accordingly, compared to cKp strains, hypermucoviscosity is the most apparent phenotypic feature of hvKp strains that usually produce hypermucoviscous colonies, thus able to form biofilms [24]. Therefore, Kp isolates can be tested for hypermucoviscosity phenotype by the string test, in which positive strains form a viscous string of more than 5 mm long when the colonies are stretched by an inoculation loop [25]. Therefore, in some reports, the hvKp variant is the so-called hypermucoid *K. pneumoniae* or hypermucoviscous *K. pneumoniae* (hmvKp) [10,12].

The virulence factors associated with the hypervirulence or hypermucoviscosity phenotype are mediated by genes commonly carried on plasmids and chromosomal genomic islands [15,26]. Such an increase in the production of surface polysaccharides is thought to protect Kp from phagocytosis and lethal serum immune factors [27]. The hypermucoviscosity-related genes include siderophore-encoding genes such as aerobactin, salmochelin and the capsular polysaccharide regulatory genes *rmpA* and *rmpA2* [28]. Although the several capsular serotypes of Kp, K1 and K2 are believed to be the main serotypes associated with the hypervirulent strains [29], genes related to fimbriae production and cell wall lipopolysaccharides may also be involved in this phenotype [8].

Based on the literature review, very few studies have been published on the characterization of the hypervirulent phenotype among commensal Kp. Therefore, this study aimed to study hypervirulence or hypermucoviscosity phenotype among gut commensal Kp isolates, determine the antimicrobial resistance profiles, and investigate the correlation between hypervirulence and both multiple antimicrobial resistance and the in vitro formation of biofilm. In addition, the study attempted to characterize the hypervirulence/hypermucoviscosity on the molecular level; thus, Kp isolates were examined for hypervirulence-related genes, including *terW*, *rmpA*, *magA*, *K2*, *uge*, *wabG*, *irp2*, *entB*, *KfuB*, *iucA*, and *iroB*.

## 2. Materials and Methods

### 2.1. Study Population, Stool Samples, and Identification of Gut Commensal Kp Isolates

The present study included 50 non-duplicate Kp stool isolates. These isolates were collected from healthy adult individuals who are essentially in good health, do not suffer from gastric diseases, and have not taken any antimicrobial therapy for no less than three months at the time of stool sample collection. The ages of the participants ranged between 24 and 60 years old. The study was conducted according to the guidelines of the Declaration of Helsinki and approved by the Ethics Committee of Heliopolis University (Ethics Code: HU.REC.H.5-2021).

The stool samples were collected in sterile screw-capped containers, transported to the microbiology laboratory, kept at 4 °C to keep the bacterial diversity and composition, and processed shortly on the same collection day. Stool culture was performed according to the method of Stanley et al. (2018) as follows [30]. The stool sample was mixed with sterile normal saline, and the stool suspension was cultured on MacConkey agar medium and incubated at 37 °C for 16 h. The lactose fermenting colonies with the typical mucoid appearance suggestive of Kp bacteria were picked and subjected to further identification using standard microbiological methods, including Gram-staining and biochemical reactions. The biochemical tests included TSI Test (Triple Sugar Iron Agar) and IMViC (Indole, Methyl red, Voges-Proskauer, and Citrate tests). Identification was confirmed by Vitek 2 automated system (bioMe’rieux, Marcy l’E´toile, France). Kp isolates were stored in glycerol stock media at −20 °C.

### 2.2. Phenotypic Assay for Identification of Hypermucoviscosity Character

The string test identified the hmvKp phenotype according to Pomakova et al. (2012) [3]. A standard bacteriologic loop was used to stretch a string from the produced colonies on MacConkey agar. A positive hypermucoviscosity phenotype was identified by forming a viscous thread-like string of more than or equal to 5 mm in length. The isolate that showed a thread-like string length of less than 5 mm was considered negative for the hypermucoviscosity phenotype or non-hmvKp.

### 2.3. Antimicrobial Susceptibility Testing and Screening for Extended-Spectrum β-Lactamase (ESBLs) Producers

The susceptibility of Kp isolates to diverse classes of antimicrobial agents was determined using the Kirby–Bauer disc diffusion method on Mueller–Hinton agar following the Clinical and Laboratory Standards Institute guidelines [31]. The 13 discs of antimicrobial agents included in this study were Oxoid (UK) products and represented eight different classes of antimicrobials. The tested antimicrobial discs were gentamicin (CN) 10 μg, amikacin (AK) 30 μg, amoxicillin-clavulanic acid (AMC) 30 μg, piperacillin-tazobactam (PTZ) 110 μg, cefotaxime (CTX) 30 μg, ceftazidime (CAZ) 30 μg, aztreonam (ATM) 30 μg, tetracycline (TE) 30 μg, ertapenem (ETP) 10 μg, imipenem (IPM) 10 μg, meropenem (MPM) 10 μg, ciprofloxacin (CIP) 5 μg, and colistin sulfate (CT) 10 μg. The results interpretation was according to the CLSI 2018 breakpoints. *Escherichia coli* ATCC25922 was used as a control strain. Screening for ESBLs producers among isolates was performed by a double-disc synergy test. Mueller–Hinton agar was inoculated with the standard inoculum of the test isolate equivalent to 0.5 McFarland. The ceftazidime (30 μg) disc was placed on agar 15 mm from the center of the amoxicillin-clavulanic acid (20 μg/10 μg) disc. An increase in zone of diameter ≥ 5 mm for either antimicrobial agent tested in combination with clavulanate versus the zone diameter of the agent when tested alone was interpreted as ESBLs producer. Kp isolate was verified as MDR when it showed resistance to at least three different antimicrobial classes [31].

### 2.4. Quantitative Biofilm Formation Assay

Biofilm formation was assayed using the microtiter plate, crystal violet method, according to Nyenje et al. (2013) [32]. Kp isolates were cultured on nutrient agar (Oxoid, UK) and plates were incubated at 37 °C for 24 h. A few single colonies of each isolate were suspended in sterile saline and adjusted to match the 0.5 McFarland turbidity standard (equivalent to 1.5 × 10^8^ CFU/mL). A 50 μL sample of each bacterial suspension was added to a 5 mL of tryptic soya broth (TSB) and incubated at 37 °C for 24 h with shaking at 200 rpm. The cultures were then diluted at 1:100 in TSB broth and an aliquot of 200 μL of diluted bacterial suspension was transferred into the microtiter plate wells in triplicates. In each plate, negative control wells contained only 200 μL of uninoculated TSB broth. The plates were incubated statically under aerobic conditions overnight at 37 °C for 24 h. After incubation, the plate content was discarded in the drain under running water to dump planktonic cells and media; then, excess planktonic cells were washed three times with 200 µL of sterile normal saline to remove the unbound bacteria. The plates were left to dry, and then 150 μL crystal violet solution (0.1%) was added to each well and left for 30 min. The plates were carefully shacked in a drain with running water to remove excess crystal violet, then left to dry for an hour at room temperature before solubilizing the dye-bound cells (biofilm) with 200 µL of 33% (*v*/*v*) glacial acetic acid to free all adsorbed crystal violet. The optical density (OD) of each well was measured using a microtiter plate reader spectrophotometer (Model 680, Biorad, UK) at a wavelength of 595 nm. The biofilm assay for each isolate was performed in triplicate on three independent repeats, and the average of the results of three repetitions and standard deviation was calculated. The cut-off was specified as three standard deviations above the mean OD of the negative control (ODc), which contained broth only. The results were interpreted according to the following criteria to classify the different adherent strengths as follows: if the mean of the three repeats OD readings ≤ ODc (the mean OD plus three standard deviations of the negative control) = non-adherent (or non-biofilm producer), ODc ˂ OD ≤ 2 × ODc = weakly adherent (or weak biofilm producer), 2 × ODc < OD ≤ 4 × ODc = moderately adherent (or moderate biofilm producer), and if 4 × ODc < OD = strongly adherent (or strong biofilm producer) [32]. *Staphylococcus aureus* ATCC 29213 was used as the positive control for biofilm production [33].

### 2.5. PCR-Based Molecular Study

#### 2.5.1. DNA Extraction and PCR Oligonucleotide Primers

DNA was extracted from all tested isolates using the boiling method according to Queipo-Ortuño et al. (2008) [34]. Bacterial cell suspensions in 50 μL of molecular biology-grade water were subjected to boiling at 100 °C for 10 min, followed by removal of cellular debris by centrifugation at 15,000× *g* for 30 s. The supernatant was collected and stored at −20 °C for use as template DNA in PCR assays. Aliquots of 2 μL of template DNA were used for PCR amplification. PCR oligonucleotide primers used in this study were synthesized by Invitrogen (UK). The lyophilized powder was reconstituted using nuclease-free water (Promega, Madison, WI, USA), and each primer concentration was adjusted to 10 pmol/µL. All PCR oligonucleotide primers are listed in Supplementary Table S1.

#### 2.5.2. Detection of Hypermucoviscousity Phenotype-Associated Genes

PCR assays were performed to determine the capsular serotypes for K1 or K2 and another 11 virulence encoding genes, including *iucA*, *entB*, *uge*, *magA*, *terW*, *iroB*, *kfuB*, *rmpA*, *rmpA2*, *wabG*, and *irp2*. PCR reaction mixtures were prepared in total volumes of 20 μL. Each reaction contained 1 μL of template DNA, 1 μL (equivalent to 10 pmol concentration) of each primer, and 10 μL of GoTaq^®^ Green Master 2 × Ready Mix (Promega, Madison, WI, USA). The volume was completed to 20 μL by 7 μL of nuclease-free water. The PCR amplification conditions were as follows: initial denaturation for 5 min at 95 °C, then 35 cycles of denaturing at 95 °C for 30 s, annealing for 30 s and extension at 72 °C, followed by a final extension at 72 °C for 7 min. The appropriate annealing temperature for each pair of primers and the time for the extension step for each PCR amplicon are mentioned in Table S1. DNA fragments of PCR products were detected through TAE agarose gel (1 %) (Bioline, London, UK) electrophoresis in 1 × TAE buffer (Thermo Scientific, Waltham, MA, USA). The Gene Ruler 1 kb DNA molecular weight marker (Thermo Scientific, Waltham, MA, USA) was used for sizing the PCR products.

### 2.6. Transmission Electron Microscopy (TEM) Study

Fresh bacterial broth cultures of representative isolates from hmvKp and non-hmvKp phenotypes were subjected to TEM examination for extracellular or capsular polysaccharide layer at the Electron Microscopy Unit in the Regional Centre for Mycology and Biotechnology, Al-Azhar University, Cairo, Egypt. The diameter of the extracellular or capsular polysaccharide layer was measured using ImageJ version 1.53n.

### 2.7. Statistical Analysis

Statistical analysis of biofilm formation assays was performed by the chi-square test using the statistical software SPSS (version 14.0, Chicago, IL, USA). The *p* value < 0.05 was considered statistically significant. Antimicrobial susceptibility results and the presence of virulence genes are presented as descriptive data of relative frequencies and percentages.

## 3. Results

### 3.1. Identification and Frequency of HmvKp Isolates among Stool Isolates

**Figure 1:**
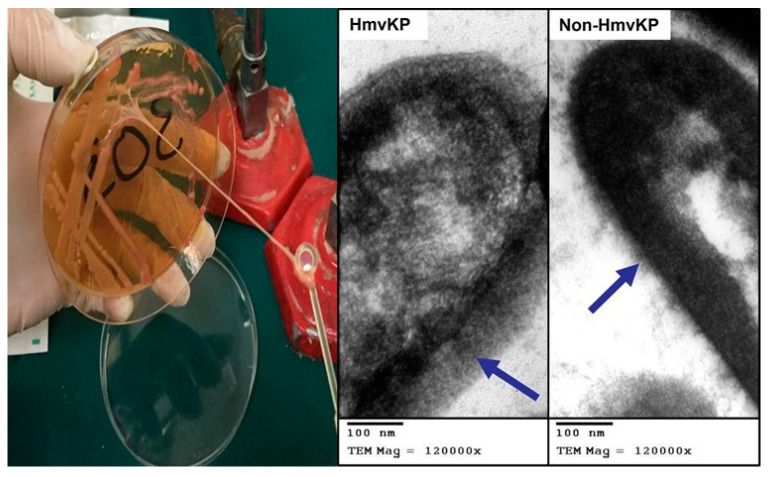
The positive result for the string test. A viscous thread-like string of more than or equal to 5 mm in length is formed, indicating positive for the hypermucoviscosity phenotype. TEM shows the surface polysaccharides in hmvKp and non-hmvKp isolated in this study. The arrows are directed to the extracellular polysaccharide layer of hmvKp, measured at an average of 87 nm, while non-hmvKp measured at an average of 22 nm.

Among the 50 Kp stool isolates recovered from stool cultures, 42% (21/50) of isolates contained hmvKp, which showed positive results for the string test as forming a viscous thread-like string of more than 5 mm in length and thus identified positive for the hmv phenotype. The frequency of non-hmvKp was 58% (29/50). The TEM study confirmed the phenotypic results, showing the extracellular polysaccharide layer in both phenotypes of an average diameter of 87 nm in hmvKp and an average diameter of 22 nm in non-hmvKp isolates (Figure 1).

### 3.2. Antimicrobial Resistance Profiles of HmvKp and Non-HmvKp Isolates

The results of the antimicrobial susceptibility testing of all Kp isolates, including the hmvKp and non-hmvKp phenotypes, against tested antimicrobial agents are presented in Table 1. The antimicrobial susceptibility profiles revealed that all isolates were MDR (50/50, 100%), resistant to at least one antimicrobial agent belonging to three different antimicrobial classes. The 50 gut Kp isolates showed high resistance rates against ciprofloxacin, cefotaxime, amoxicillin-clavulanic acid, aztreonam, and ceftazidime of 100%, 94%, 72%, 70%, and 70%, respectively. Colistin showed the highest sensitivity rate among isolates (43/50, 86%).

Concerning the antimicrobial resistance profiles in hmvKp phenotypes compared to non-hmvKp isolates, non-hmvKp isolates showed higher resistance rates than hmvKp phenotypes among the total number of resistant isolates to each antimicrobial agent, except for amikacin, amoxicillin-clavulanic acid, ertapenem, and tetracycline. In comparison, hmvKp and non-hmvKp isolates showed equal resistance rates to each ciprofloxacin and meropenem of 100% and 50%, respectively. All resistant isolates to colistin were non-hmvKp isolates; hmvKp phenotypes showed no resistance to colistin.

Screening for ESBLs production revealed that 76 % (38/50) of all Kp isolates were ESBLs producers. Among these positive ESBLs producers, non-hmvKp isolates showed higher frequencies (23/38, 60.5%) than hmvKp phenotypes (15/38, 39.5%).

### 3.3. Biofilm Formation Characters among HmvKp and Non-HmvKp Phenotypes

**Figure 2:**
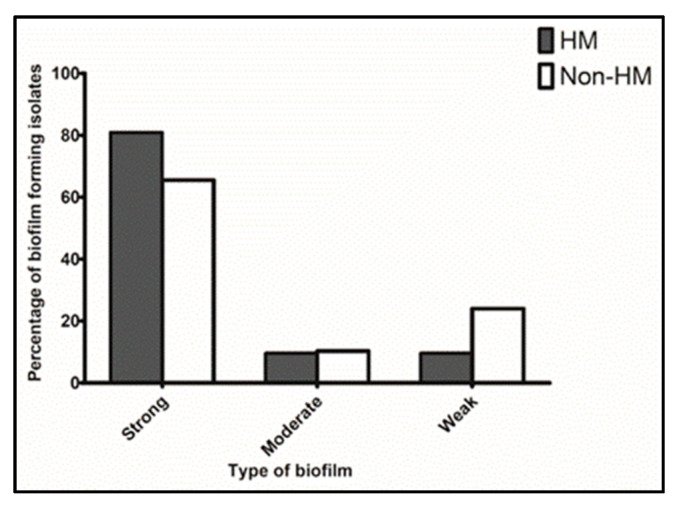
Assay of biofilm formation by hmvKp and non-hmvKp isolates. There was no significant difference between hmvKp and non-hmvKp phenotypes among each strong, moderate, and weak biofilm-forming isolates (*p* > 0.05). HM, hypermucoviscous; non-HM, non-hypermucoviscous.

Overall, there was no significant difference between the frequency of hmvKp and non-hmvKp isolates in each biofilm formation grade (*p* > 0.05) (Figure 2). In detail, high frequencies of strong biofilm-forming isolates were identified in both hmvKp phenotypes and non-hmvKp isolates than those of moderate and weak biofilm-forming ones. The frequency of strong biofilm-forming among hmvKp isolates (81%, 17/21) was higher than that determined among non-hmvKp isolates (65.5%, 19/29). On the other hand, the rate of weak biofilm formation among non-hmvKp isolates of 24% (7/29) was higher than that detected among hmvKp isolates of 9.5% (2/21). The rate of moderate biofilm-forming isolates was 10.3% (3/29) and 9.5% (2/21) among hmvKp and non-hmvKp isolates, respectively.

### 3.4. Distribution of Hypermucoviscosity Phenotype-Related Virulence Genes among HmvKp and Non-HmvKp Isolates

All isolates were examined by PCR for the presence of hypermucoviscosity phenotype-related virulence genes. Among the 50 Kp isolates, *terW* was the most frequently detected gene (98%), followed by *wabG*, *uge*, and *entB* with frequencies of 96%, 94%, and 92%, respectively. The *iroB*, *rmpA*, *rmpA2*, and *magA* genes were not detected in any isolates (Table 2).

Among hmvKp isolates, the genes *terW*, *wabG*, *entB*, and *uge* were the most prevalent, with frequencies of 100%, 90.5%, 90.5%, and 85.7%, respectively. In non-hmvKp isolates, the most frequent genes were *wabG* and *uge*, which were detected in all isolates (100%), followed by each *terW* and *irp2* (96.6%) and then *entB* with a frequency of 93.1%. PCR-based molecular screening for K capsular serotype revealed that 30% (15/50) of Kp isolates belonged to capsular serotype K2 with 13 out of the 15 isolates being hmvKp phenotypes. In contrast, no isolates belonged to K1 (*magA* gene) capsular serotype. Two non-hmvKp isolates belonged to the K2 capsular serotype (Table 2).

The co-presence of genes *terW*, *KfuBc*, *wabG*, *uge*, *irp2*, and *entB* was the most frequent genotypic profile among all isolates (26%), followed by the co-presence of *terW*, *wabG*, *uge*, *irp2*, and *entB* (24%). The genotypic profiles *terW*-*KfuBc*-*wabG*-*uge*-*irp2*-*entB*-*K2*, *terW*-*wabG*-*uge*-*entB*, and *terW*-*wabG*-*uge*-*entB*-*K2* were frequent among hmvKp isolates with a frequency of 14.3%. Among non-hmvKp isolates, the genotypic profile *terW*-*KfuBc*-*wabG*-*uge*-*irp2*-*entB* was the most prevalent, followed by *terW*-*wabG*-*uge*-*irp2*-*entB* with frequencies of 44.8% and 41.4%, respectively (Table 3).

## 4. Discussion

The hvKp (or hmvKp) is a ubiquitous superbug that causes hospital- and community-acquired invasive infections, including pyogenic liver abscess, meningitis, and endophthalmitis [35,36,37,38,39,40]. The hmvKp-caused bacteremia resulted in a mortality rate of 35% [25]; in a case series of 12 patients with hmvKp, five patients with bacteremia died [41]. Hypermucoviscous or hypervirulent are alternatively used in the literature [36,40]. Notably, a high prevalence of hvKp-related pyogenic liver abscess was documented in Asian countries such as Taiwan, China, South Korea, and Iran [2,19,42,43]. However, it is unclear whether the emergence of hvKp in Asia is due to genetic predisposition in these populations, environmental reasons, or other unidentified factors [9]. This research attempted to study the hypermucoviscosity phenotype and associated genes among the gut commensal Kp isolates from healthy Egyptian individuals.

The present research isolated 50 gut commensal Kp isolates from stool samples of healthy Egyptians. These isolates were examined for the hypermucoviscosity phenotype by string test, a standard method for determining hypermucoviscosity [44]. A positive string test typically indicates hypermucoviscosity or hypervirulence for Kp [45]. In the current study, 42% (21/50) of Kp isolates were identified as hmvKp, as they showed a positive string test result by forming a viscous string of more than 5 mm. In comparison, the frequency of non-hmvKp isolates was 58% (29/50). However, the ability of the string test to predict clinical features associated with hvKp infection varies with correlation ranging from 51% [46] to as high as 90% [47], 95% [42], and 98% [36]. Accordingly, infection control practitioners may implement the string test in routine microbiological inspections in hospital wards and intensive care units [48]. It is noteworthy that the sensitivity, specificity, and accuracy of string test for identifying hvKp, compared to PCR data of encoding genes, have been assessed in various previous studies. Elbrolosy (2021) study revealed that the sensitivity, specificity, and accuracy of string test are 95.2%, 79.3%, and 86%, respectively, with a positive predictive value of 76.9% and a negative predictive value of 95.8% [49]. In addition, Russo and Gulick (2019) demonstrated string test results to identify hvKp with a specificity of 89%, a sensitivity of 91%, and an accuracy of 90% [27]. Moreover, in a study, the string test showed 32.7% positive predictive value, 97.2% negative predictive value, 63.9% specificity, and 90.5% sensitivity [45]. Remarkably, the low specificity percentage of the string test is particularly problematic in low-prevalence areas, such as Egypt. On the other hand, many studies have reported clinical isolates of non-hmvKp that caused pyogenic liver abscesses [50,51,52]. Consequently, since the string test has a wide range of stated sensitivity and specificity values, hvKp should be identified based on the hypermucoviscosity feature and the detection of other virulence factors [52].

The hvKp with excessive polysaccharide production can infect healthy individuals, causing life-threatening infections [29]. The hvKp utilizes diverse virulence factors for pathogenesis and survival, including siderophores, enhanced capsule formation, lipopolysaccharides, and fimbriae production [36]. It has been recently revealed that cKp strains could readily acquire the hypervirulence phenotype by obtaining the virulence-carrying plasmids by conjugation; such an event results in a sharp increase in the incidence of hvKp infections [44]. In the current study, Kp isolates were analyzed for certain genes of significant importance in expressing different virulence factors, including *magA*, *wzy*, *kfuBC*, *entB*, *iroB*, *irp2*, *terW*, *wabG*, *uge*, *iucA*, *rmpA*, and *rmpA2*.

Currently, capsular typing is a widely used technique for typing Kp. Among no less than 78 capsular serotypes, K1 and K2 capsular serotypes lack sugar residues in the capsule, which are recognized by macrophage lectin receptors; therefore, these Kp serotypes are protected from phagocytosis [9]. Remarkably, these capsular serotypes are more prevalent in Kp invasive infections [1]. The mucoviscosity-associated gene *magA* is restricted explicitly to capsular serotype K1. In addition, the *wzy* gene is the serotype-specific gene for the K2 capsular serotype [1]. In the current study, PCR-based molecular screening for K1 and K2 capsular serotypes revealed that 30% (15/50) of Kp isolates belonged to capsular serotype K2 (harboring *wzy* gene), with 86.7% (13/15) of the K2 isolates being hmvKp phenotypes, which represent 61.9% of all hmvKp isolates. None of the Kp isolates have been identified as the K1 capsular type attributable to the absence of the *magA* gene [53,54]. However, it was reported that the *magA* gene is confined to liver abscesses and linked to hypermucoviscosity and phagocytosis resistance [2,55,56,57]. In addition, other studies revealed the significant contribution of *magA* in serious Kp infections, including bacteremia, pneumonia, septicemia, and liver and lung abscesses [53,58,59]. In this study, two non-hmvKP isolates belonged to the K2 capsular serotype. These findings are consistent with other studies that mostly concluded that K2 is the most frequent capsular serotype among hmvKp isolates compared to K1 [60,61]. Further, these data agreed with other studies that revealed the absence of the *magA* gene in all hmvKp isolates [62,63].

The higher capsule production Is a crucial virulence factor that can protect bacteria from the host immune response [9]. However, K1 and K2 capsular types do not fully account for hypervirulence; thus, K1 and K2 capsules commonly co-occur with other virulence factors [64]. For instance, in serotype K1, the genes responsible for the expression of aerobactin and salmochelin *iuc* and *iro*, respectively, are located on the same plasmid as the genes for mucoid phenotype *rmpA*. There are three forms of the hypermucoviscosity-related gene *rpmA* (regulator of mucoid phenotype A); plasmid-carried genes p-*rpmA* and p-*rpmA2*, and chromosomal-mediated gene c-*rpmA* [23,24]. The gene *rpmA* coordinates the capsule and hypermucoviscosity, while c-*rpmA* regulates only the capsule formation [9]. Consistent with other studies, the *rmpA* gene was absent in the current study isolates [63,65]. However, several studies reported the presence of the latter genes in both hmvKp and cKp strains [66]. This inconsistency could be attributed to the variability in the study condition, including the source and the colonization niche of isolates, Kp isolates in the current study are gut commensals, or the study population. The absence of the already known essential genes related to mucoviscosity such as *magA* and *rmpA* in hmvKp isolates proposes the multifactorial complexity of the hypermucoviscosity or hypervirulence phenotype. Thus, further studies should be carried out to verify the hypermucoviscosity-related virulence factors among clinical and commensal Kp from different niches.

Kp produces and secretes siderophores that are essential virulence factors for the hvKp, compared to cKp. The siderophores scavenge ferric iron from the environment. These siderophores include aerobactin (encoded by *iuc* genes), salmochelin (encoded by *iro* genes), enterobactin (encoded by *entB* genes), and yersiniabactin (encoded by *irp* genes) [1,52]. In the present study, the most prevalent siderophores encoding genes in Kp isolates were *entB* (92%), followed by *irp2* (72%); however, *iucA* was only detected in one isolate. Most hmvKp isolates (90.5%) harbored *entB*, and 96.6% of non-hmvKp carried *irp2* gene. Therefore, *entB* and *irp2* genes are the most prominent siderophores encoding genes in hmvKp and non-hmvKp, respectively. These findings agreed with other studies that reported that *entB* was the most prevalent siderophore among their isolates [60,65,66]. In addition, these data are consistent with other studies that revealed the aerobactin encoding gene is less detected than that encoding enterobactin [63,66]. *KfuBC*, which encodes for the iron uptake system, was detected in 44% of the present study isolates, similar to previous studies [27,61,67]. Indeed, several iron transport mechanisms in hvKp are rational and complementary since these mechanisms allow hvKp to operate in various organs and/or microenvironments during infection [39]. In addition, the presence of any of these siderophores is essential and contributes to the hypermucoviscosity phenotype.

The correlation between bacterial pathogenicity and tellurite resistance has been revealed. Tellurite is a very toxic substance for bacteria, so it is used in clinical pathogen screening [68]. In the current study, the tellurium resistance gene *terW* was detected in 98% of Kp isolates, and all hmvKp isolates harbored this gene. This is unlike the study by Passet and Brisse (2015), which reported only 25% of their study isolates of diverse phylogeny were positive for the same gene [69]. Compared to Passet and Brisse (2015), the varying carriage rate of *terW* in hmvKp in the current study (100%) may be attributed to different sources of isolates, or it may be due to the phylogenetic diversity of Kp in their research. However, it indicates the less significant role of tellurite resistance in the hypermucoviscosity trait.

Various genes may control the production of capsule polysaccharides (K antigen), including the *uge* gene. The *uge* gene encodes uridine diphosphate galacturonate 4-epimerase, which is crucial for the virulence of Kp to be capable of causing urinary tract infections, pneumonia, and sepsis. In addition, the *wabG* gene is essential in the biosynthesis of core lipopolysaccharides. These genes are involved in Kp invasion, colonization, and pathogenicity [70]. The results of the current study revealed that 96% and 94% of the isolates harbored the genes involved in lipopolysaccharide biosynthesis, *wabG* and *uge*, respectively. Notably, all non-hmvKp isolates (100%) showed the carriage of both genes. In comparison, 90.5% and 85.7% of hmvKp isolates harbored *wabG* and *uge*, respectively, indicating the likely hypervirulence of these Kp gut commensal isolates; thus, they are a potential source of severe invasive infections. The frequency of the *uge* gene was consistent but a little higher than other studies that reported the occurrence of this gene, with frequencies ranging between 38.2% and 86% among Kp isolates [62,63,66].

Forming biofilm is a crucial step in the pathogenesis of Kp-caused diseases. Biofilm increases resistance to environmental stresses and antimicrobial agents and serves as a reservoir for subsequent gene exchange. Many virulence factors help Kp to produce biofilm, either directly by encouraging more remarkable adhesion or biofilm maturation and indirectly by preventing biofilm formation by competing bacteria in their colonization niche [71]. In the current study, biofilm formation assays revealed that most hvKp isolates (75%) were strong biofilm producers, consistent with a previous study in Iraq [72]. Additionally, Nirwati et al. (2019) reported that 85.63% of Kp isolates were biofilm producers, and 64.7% of these isolates were strong biofilm producers, according to a related study by Hassan et al. (2011) [73,74]. Our data also showed no differences in the biofilm formation of hmvKp and non-hmvKp phenotypes among each strong, moderate, and weak biofilm-forming isolates; these results were consistent with clinical isolates investigated in Soto et al.’s study [75,76].

Based on the previous information on the presence of virulence factors encoding genes, all tested virulence encoding genes can simultaneously enhance the potential pathogenicity of commensal Kp isolates. Remarkably, all study Kp isolates (100%) from healthy individuals co-harbored more than one virulence-encoding gene. For instance, 44.8% of non-hmvKp isolates carry virulence genes, including *ter*, *KfuBc*, *wabG*, *uge*, *irp2*, and *entB*. Furthermore, Remya et al.’s (2019) study revealed that one or more virulence genes exist among 90% of Kp clinical isolates [66]. These findings highlight the high risk of commensal hvKp harboring multiple virulence genes in healthy individuals and indicate the potential role of commensal Kp in disease conditions. Strakova (2021) tried to summarize the definition of hvKp strains by detecting p-*rmpA*, p-*rmpA2*, *iroB*, *iucA*, and *peg-344* genes; however, the selection of genetic markers used for the characterization of hvKp is not clear and is complicated [1]. Indeed, according to the data of the current study and published studies in the literature, many diverse aspects can contribute to hypermucoviscosity and/or hypervirulence trait of Kp, including different virulence factors and the colonization niche of Kp strains, such as the gut, lung, or urinary tract system, in addition to commensal or pathogenic. Furthermore, the candidate genes are of variable importance for hypermucoviscosity and hvKp strains. However, this study is an in vitro attempt to study hypermucoviscosity controlling factors; thus, in the actual in vivo situation, such as the gut environmental niche, the expression of different virulence factors genes may affect these phenotypes. Therefore, investigating in vivo expression of virulence factors encoding genes related to these phenotypes in various environmental niches is warranted.

The spread of MDR hvKp strains represents a critical public health challenge worldwide [23,39,40,77]. The current study finding that all Kp isolates were MDR is consistent with the study of Huynh et al. (2020) [78]. The antibiogram of Kp isolates showed increased resistance toward cephalosporins and fluoroquinolones [79]. In the present study, hvKp were highly resistant to ciprofloxacin (100%), cephalosporins (70–94%), and aztreonam (70%). These data are consistent with other studies that showed high resistance patterns of the Kp isolates to ceftazidime and ciprofloxacin [80,81]. The co-resistance pattern to cephalosporins and quinolones may be attributed to ESBLs encoding genes, and resistance genes for quinolones are present on the same plasmids [82]. However, this is consistent with Karimi’s (2021) study, where 86% of the isolates demonstrated sensitivity to colistin [83].

The present study percentage of ESBL-producing Kp was 38%. Other studies showed different rates of ESBL-producing Kp ranging from 9.6% to 81.1% [84]. The difference in ESBL-producing Kp percentages may be explained by the variable degree of exposure of beta-lactam antibiotics to this organism or because of the varying degree of transferability of plasmids that carry the ESBL genes. The growing prevalence and genetic similarity of ESBL-producing Kp and carbapenemase-producing Kp in humans and chickens provide a public health risk and require more careful use of antibiotics in chicken farms to prevent the spread of both ESBL and carbapenem resistance in Kp [14,85]. Indeed, carbapenem-resistant Kp is a “serious concern” for the World Health Organization [86]. In the current study, the resistance rates of hvKp to carbapenems were 54%, 42%, 48%, and 14% to each imipenem, ertapenem, meropenem, and colistin, respectively. Many clinically used antimicrobials are inactive against β-lactamases, so the optimal treatment of carbapenem-resistant Kp is still unknown [27,87]. The high resistance profile and 100% frequency of MDR in Kp isolates in this study can be explained by the acquisition of antimicrobial resistance genes carried on mobile genetic elements by horizontal gene transfer mechanisms among the gut bacterial community [23,39,88]. The other possible reason is the extensive administration and misuse of antimicrobial drugs due to their availability for use without a prescription in Egypt, in addition to poor patient compliance by administering incomplete dose regimens. Furthermore, the results of the present study showed that the percentages of the resistant isolates to various tested antimicrobials were higher among non-hmvKp isolates than hmvKp isolates. That could be attributed to the ease of acquiring antimicrobial resistance genes by the non-hmvKp strains due to the lack of the hypermucoviscosity trait.

The strength of the current study is that, as far as we know, this is the first report highlighting the hypermucoviscosity or hypervirulence trait and MDR features in gut commensal Kp isolated from healthy individuals in Egypt. This represents a potential source of severe invasive infections in humans. Accordingly, this study might have important implications for future research on gut commensal Kp bacteria virulence and antimicrobial susceptibility. However, one of the limitations of this study is the small sample size. Additionally, whole genome sequencing is crucial for better understanding the elements underlying the hypervirulence or hypermucoviscous phenotypes; however, the resources and high cost were a constraint. The hypermucoviscosity-related virulence factors were studied by in vitro experiments, which only partially reflect the effect of the nature of the gut and its physiological characteristics on the behavior of gut microbiota. Therefore, in vivo study and RT-PCR analyses are necessary to assess the level of expression of the virulence genes in gut Kp isolates, which could accurately disclose the underlying genetic mechanisms of hvKp strains and how these isolates behave in the gut niche.

## 5. Conclusions

The current study shows a significant spread of MDR hvKp strains among the gut commensal Kp isolates, which can exaggerate the danger of invasive infections. Furthermore, this commensal hmvKp is MDR which limits the clinically used antimicrobials and, consequently, represents a significant challenge in healthcare settings. Diverse virulence factors are suggested to contribute to hypermucoviscosity or hypervirulence phenotypes with varying significance. For instance, any of the siderophores in Kp could be essential and contribute to the hypermucoviscosity phenotype. Tellurite resistance has a less significant role in the hypermucoviscosity phenotype. The absence of the commonly related genes to this phenotype, *magA* and *rmpA*, suggests the complex multifactorial hypermucoviscosity trait. Thus, further in vitro and in vivo studies of virulence factors contributing to hypermucoviscosity or hypervirulence phenotype, in both clinical and commensal Kp strains, in different colonization niches are essential. Moreover, more studies are urgently required concerning the determination, pathogenesis, prevention, and treatment of MDR hvKp infection.

## Figures and Tables

**Table 1:** Antimicrobial susceptibility frequencies of hmvKp and non-hmvKp isolates.

Antimicrobial Agent	Susceptibility			HmvKp		Non-HmvKp	
		No.	% ^1^	No.	% ^2^	No.	% ^2^
Gentamicin	S	27	54%	10	37%	17	63%
	R	23	46%	11	47.8%	12	52.2%
Amikacin	S	37	74%	14	37.8%	23	62.2%
	R	13	26%	7	53.8%	6	46.2%
Amoxicillin-clavulanic acid	S	14	28%	2	14.3%	12	85.7%
	R	36	72%	19	52.8%	17	47.2%
Piperacillin-Tazobactam	S	26	52%	10	38.5%	16	61.5%
	R	24	48%	11	45.8%	13	54.2%
Cefotaxime	S	3	6%	2	66.7%	1	33.3%
	R	47	94%	19	40.4%	28	59.6%
Ceftazidime	S	15	30%	7	46.7%	8	53.3%
	R	35	70%	14	40%	21	60%
Aztreonam	S	15	30%	6	40%	9	60%
	R	35	70%	15	42.9%	20	57.1%
Imipenem	S	23	46%	9	39%	14	61%
	R	27	54%	12	44.4%	15	55.6%
Meropenem	S	26	52%	9	34.6%	17	65.4%
	R	24	48%	12	50%	12	50%
Ertapenem	S	29	58%	10	34.5%	19	65.5%
	R	21	42%	11	52.4%	10	47.6%
Ciprofloxacin	S	0	0%	0	0%	0	0%
	R	50	100%	21	42%	29	58%
Tetracycline	S	20	40%	5	25%	15	75%
	R	30	60%	16	53.3%	14	46.7%
Colistin	S	43	86%	21	48.8%	22	51.2%
	R	7	14%	0	0%	7	100%
ESBLs producer		38	76% ^3^	15	39.5%	23	60.5%

^1^ Percentage correlated to the total number of isolates (50 isolates). ^2^ Percentage correlated to the total number of sensitive or resistant isolates to each antimicrobial agent. ^3^ Percentage correlated to the total number of ESBLs producers (38 isolates). S: sensitive; R: resistant.

**Table 2:** Prevalence of hypermucoviscosity phenotype-related virulence genes among gut commensal Kp isolates.

Target Gene	Total No. (%) ^1^	HmvKp (*n* = 21)			Non-HmvKp (*n* = 29)		
		No.	% ^2^	% ^3^	No.	% ^2^	% ^4^
For Capsular Serotype							
serotype K1 (*magA*)	0	0	0	0	0	0	0
serotype K2 (*wzy*)	15 (30%)	13	86.7%	61.9%	2	13.3%	6.9%
Virulence Genes							
*terW*	49 (98%)	21	42.9%	100%	28	57.1%	96.6%
*wabG*	48 (96%)	19	39.6%	90.5%	29	60.4%	100%
*uge*	47 (94%)	18	38.3%	85.7%	29	61.7%	100%
*entB*	46 (92%)	19	41.3%	90.5%	27	58.7%	93.1%
*irp2*	37 (74%)	9	24.3%	42.9%	28	75.7%	96.6%
*KfuBc*	22 (44%)	8	36.4%	38.1%	14	63.6%	48.3%
*iucA*	1 (2%)	1	100%	4.8%	0	0%	0%
*iroB*, *rmpA*, *rmpA2*	0	0	0%	0%	0	0%	0%

^1^ Percentage correlated to the total number of *K. pneumoniae* isolates (*n* = 50). ^2^ Percentage correlated to the total number of *K. pneumoniae* isolates harboring the gene. ^3^ Percentage correlated to the total number of HMV isolates (*n* = 21). ^4^ Percentage correlated to the total number of non-HMV isolates (*n* = 29).

**Table 3:** Genotypic profiles of virulence genes among hmvKp and non-hmvKp isolates.

Genotypic Profile	Total No. (%) ^1^	HmvKp (*n* = 21)		Non-HmvKp (*n* = 29)	
		No.	% ^2^	No.	% ^3^
*terW-KfuBc-wabG-uge-irp2-entB*	13 (26%)	0	0%	13	44.8%
*terW-wabG-uge-irp2-entB*	12 (24%)	0	0%	12	41.4%
*terW-wabG-uge-entB*	3 (6%)	3	14.3%	0	0%
*terW-wabG-uge-entB-K2*	3 (6%)	3	14.3%	0	0%
*terW-KfuBc-wabG-uge-irp2-entB-K2*	3 (6%)	3	14.3%	0	0%
*terW-wabG-uge-irp2-entB*	2 (4%)	2	9.5%	0	0%
*terW-wabG-uge-irp2-entB-K2*	2 (4%)	2	9.5%	0	0%
*KfuBc-wabG-uge-entB*	1 (2%)	0	0%	1	3.4%
*terW-KfuBc-wabG-uge-entB*	1 (2%)	1	4.8%	0	0%
*terW-wabG-uge-irp2*	1 (2%)	0	0%	1	3.4%
*terW-wabG-uge-irp2-entB-K2*	1 (2%)	0	0%	1	3.4%
*terW-wabG-uge-irp2-K2*	1 (2%)	0	0%	1	3.4%
*terW-uge-entB*	1 (2%)	1	4.8%	0	0%
*terW-KfuBc-uge-entB-K2*	1 (2%)	1	4.8%	0	0%
*terW-KfuBc-wabG-irp2-entB*	1 (2%)	1	4.8%	0	0%
*terW-KfuBc-wabG-K2*	1 (2%)	1	4.8%	0	0%
*terW-KfuBc-wabG-uge--K2*	1 (2%)	1	4.8%	0	0%
*terW-wabG-irp2-entB-K2*	1 (2%)	1	4.8%	0	0%
*terW-wabG-uge-entB-K2-iucA*	1 (2%)	1	4.8%	0	0%

^1^ Percentage correlated to the total number of Kp isolates (*n* = 50). ^2^ Percentage correlated to the total number of HMV isolates (*n* = 21). ^3^ Percentage correlated to the total number of non-HMV isolates (*n* = 29).

## Data Availability

Data are available upon request.

## Supplementary Materials

The following supporting information can be downloaded at https://www.mdpi.com/article/10.3390/microorganisms11030704/s1, Table S1: DNA sequences of oligonucleotide PCR primers.
