# Supplementary material for: Occurrence and Molecular Study of Hypermucoviscous/Hypervirulence Trait in Gut Commensal K. pneumoniae from Healthy Subjects
Source: Microorganisms. 2023 Mar 9;11(3):704. doi: 10.3390/microorganisms11030704 (PMC10059952; doi:10.3390/microorganisms11030704)
Supplement: Supplementary file 1 [file microorganisms-11-00704-s001.zip › microorganisms-2242571-supplementary.pdf]

**Supplementary Materials**

**Table S1.** DNA sequences of oligonucleotide PCR primers.

| Target gene and function | Sequence (5' – 3') | Amplicon size (bp) | Ta [1] / extension time | Source |
|---|---|---|---|---|
| *magA*: mucoviscosity associated gene A: (serotype K1); for capsular serotyping | F: GGTGCTCTTTACATCATTGC<br>R: GCAATGGCCATTTGCGTTAG | 1283 bp | 53 °C / 90 s | [35] |
| capsular serotype K2 gene (*wzy*): plays a vital role in the synthesis of the lipopolysaccharide (LPS); for capsular serotyping | F: GACCCGATATTCATACTTGACAGAG<br>R: CCTGAAGTAAAATCGTAAATAGATGGC | 641 bp | 57 °C / 50 s | [35] |
| *kfuBC*: uptake of ferric iron | F: GAAGTGACGCTGTTTCTGGC<br>R: TTTCGTGTGGCCAGTGACTC | 797 bp | 57 °C / 1 min | [35] |
| *entB*: enterobactin | F: GATGAAGACGATACCGTGC<br>R: ACCGAATCCAGACCGTAGTC | 389 bp | 53 °C / 30 s | [36] |
| *iroB*: salmochelin siderophore biosynthesis | F: ATCTCATCATCTACCCTCCGCTC<br>R: GGTTCGCCGTCGTTTTCAA | 235 bp | 59 °C / 30 s | [36] |
| *irp2*: yersiniabactin siderophore biosynthesis | F: GCTACAATGGGACAGCAACGAC<br>R: GCAGAGCGATACGGAAAATGC | 230 bp | 59 °C / 30 s | [36] |
| *terW*: tellurite resistance (pLVPK-derived genetic loci) | F: ATGCAATTAAACACCAGACAG<br>R: CTCATTCTCTTGAGTGTTTTC | 239 bp | 51°C / 30 s | [37] |
| *wabG*: involved in the biosynthesis of the outer core lipopolysaccharide | F: ACCATCGGCCATTTGATAGA<br>R: CGGACTGGCAGATCCATATC | 683 bp | 56 °C / 50 s | [38] |
| *uge*: uridine diphosphate galacturonate 4-epimerase, responsible for the biosynthesis of the capsule and smooth lipopolysaccharide | F: TCTTCACGCCTTCCTTCACT<br>R: GATCATCCGGTCTCCCTGTA | 534 bp | 57 °C / 40 s | [38] |
| *iucA*: aerobactin | F: AATCAATGGCTATTCCCGCTG<br>R: CGCTTCACTTCTTTCACTGACAGG | 239 bp | 59 °C / 30 s | [36] |
| *rmpA*: extracellular polysaccharide synthesis | F: ACTGGGCTACCTCTGCTTCA<br>R: CTTGCATGAGCCATCTTTCA | 535 bp | 57 °C / 50 s | [35] |
| *rmpA2*: extracellular polysaccharide synthesis | F: GTGCAATAAGGATGTTACATTA<br>R: GGATGCCCTCCTCCTG | 430 bp | 50 °C / 40 s | [36] |

[1] Ta: annealing temperature.
